# Identification of four secretory phospholipase A_2_s in a lepidopteran insect, *Acrolepiopsis sapporensis*, and their functional association with cellular immune responses

**DOI:** 10.3389/fendo.2023.1190834

**Published:** 2023-06-23

**Authors:** Md Tafim Hossain Hrithik, Jooan Hong, Yonggyun Kim

**Affiliations:** Department of Plant Medicals, Andong National University, Andong, Republic of Korea

**Keywords:** eicosanoid, PLA2, immunity, nodulation, RNAi, *Acrolepiopsis sapporensis*

## Abstract

**Background:**

Eicosanoids are a group of the oxygenated C20 polyunsaturated fatty acids and play crucial roles in mediating various insect physiological processes. Catalytic activity of phospholipase A_2_ (PLA_2_) provides an initial substrate, arachidonic acid (AA), for subsequent eicosanoid biosynthesis.

**Results:**

This study identified four different secretory PLA_2_ (*As-PLA_2_A*–*As-PLA_2_D*) genes encoded in the Asian onion moth, *Acrolepiopsis sapporensis*. A phylogenetic analysis indicated that *As-PLA_2_A* and *As-PLA_2_D* are clustered with Group III PLA_2_s while *As-PLA_2_B* and *As-PLA_2_C* are clustered with Group XII and Group X PLA_2_s, respectively. Expression levels of these PLA_2_ genes increased along with larval development, especially in the fat body. A bacterial immune challenge upregulated the basal expression levels of the four PLA_2_ genes, which resulted in significant increases of the PLA_2_ enzyme activity. The enzyme activity was susceptible to a calcium chelator or reducing agent, suggesting Ca^2+^ dependency and disulfide linkage required for the catalytic activities of the secretory type of PLA_2_s. In addition, the PLA_2_ activity was also susceptible to bromophenacyl bromide (BPB), a specific inhibitor to sPLA_2_, but not to intracellular PLA_2_ inhibitors. An addition of BPB to the immune challenge significantly prevented hemocyte-spreading behavior of *A. sapporensis*. BPB treatment also suppressed a cellular immune response measured by hemocyte nodule formation. However, the immunosuppression was significantly rescued by the AA addition. To determine the PLA_2_(s) responsible for the immunity, individual RNA interference (RNAi) treatments specific to each of the four PLA_2_s were performed. Injection of gene-specific double-stranded RNAs caused significant reductions in the transcript level in all four PLA_2_s. In all four PLA_2_s, the RNAi treatments prevented the cellular immune response even after the immune challenge.

**Conclusion:**

This study reports four secretory PLA_2_s encoded in *A. sapporensis* and their function in mediating cellular immunity.

## Introduction

Polyunsaturated fatty acids (PUFAs) are preferentially associated with phospholipid (PL) components of biological membranes. PUFAs are associated with biomembrane properties of fluidity and thickness (1), while they occur in much lower proportions in neutral and energy-storage lipids such as triacylglycerols (2, 3). Phospholipase A_2_ (PLA_2_) catalyzes the hydrolysis of PUFAs from PLs (4, 5). PLA_2_s exert a wide range of biological actions, such as dietary PL digestion, remodeling of lipid bilayer of cellular and subcellular membranes, signal transduction, and immunity (6). These diverse functions are supported by at least 16 groups (I-XVI) of PLA_2_s among biological systems (7). The six main types of PLA_2_ enzymes are the secreted (sPLA_2_ including I–III, V, IX–XIV, and XVI groups), cytosolic (cPLA_2_, IV), calcium-independent (iPLA_2_, VI), platelet-activating factor acetyl hydrolase (PAF-AH, VII, VIII), also known as lipoprotein-associated PLA_2_ (Lp-PLA_2_, VIIA), lysosomal PLA_2_ (LPLA_2_, XV), and adipose PLA_2_ (AdPLA) (8).

PLA_2_ acts as the first biochemical step in eicosanoid signaling by releasing certain PUFAs from biomembrane PLs, specifically C18 or C20 PUFAs that can serve as precursors to eicosanoid signaling responsible for defending insects from invading pathogens (9, 10). Park and Kim (11) first revealed the broad biological significance of PLA_2_ actions in insect immunity with their discovery that eicosanoid treatments rescued beet armyworms, *Spodoptera exigua*, from lethal bacterial, *Xenorhabdus nematophila*, infections. They also document the bacterial action of inhibiting PLA_2_ activity required to release PUFAs from PLs for eicosanoid biosynthesis. Park et al. (12) reported that an organic extract of the bacterial broth used to grow *X. nematophila* inhibits PLA_2_s from insect, prokaryote, and vertebrate sources. Later, nine secondary metabolites containing phenyl propylene skeletons including benzylideneacetone were chemically identified from the bacterial culture broth. These compounds are competitive inhibitors of PLA_2_ (13).

Due to the absence or near-absence of arachidonic acid (AA) in insect PLs, it was not immediately clear that eicosanoids signal insect immunity. Mammals maintain substantial proportions (~13.1%) of AA in PLs, from which it can be hydrolyzed for eicosanoid biosynthesis (14). Insects tend to maintain very low proportions of AA (often no more than trace amounts) and high proportions of linoleic acid (LA; 18:2n-6) in cellular PLs, which may help reduce oxidative damage to cellular PLs (15). Hasan et al. (10) demonstrated AA biosynthesis from LA in *S. exigua* following immune challenge via sequential elongase and desaturase activities. Indeed, the bacterial infections led to increased proportions of AA from undetectable in naïve *S. exigua* larvae to 0.20% after bacterial infections (10, 16). The increased AA was converted into various eicosanoids, such as prostaglandins (PGs) and epoxyeicosatrienoic acids that mediate cellular and humoral immune responses (17–19). The LA metabolites including C18 lipoxins signal immune reactions to insect pathogens such as malarial parasites in mosquitoes (20) and bacterial pathogens in *S. exigua* (21). Although little information is available about the role of eicosanoids in insect nervous systems, eicosanoids act as intracellular signals by stimulating neuropeptides such as calcitonin gene–related peptide release in mammalian trigeminal ganglion neurons, which are cranial nerves responsible for sensation and motor functions in the face (22). These findings suggest that PLA_2_ is an essential step in coordinating immune and nervous signalings in insects.

The Asiatic onion moth, *Acrolepiopsis sapporensis*, is native to Asia, including China, Mongolia, Russia, Japan, and Korea, and its larvae infest a high-value crop, the Welsh onion (23). Despite serious concerns of massive economic losses due to *A. sapporensis* infestation, very little research into identifying and developing molecular targets for development of new, effective management technologies has been reported for this costly pest insect. Insect immunity is a powerful and effective defense against a wide range of entomopathogens, which may be an effective target for novel *A. sapporensis* control measures. Because PLA_2_ is the first biochemical step in eicosanoid signaling targeting the gene encoding this enzyme, it would be a workable strategy to develop novel control agent(s) against *A. sapporensis*. Here, we suggest that application of immunosuppressants by targeting PLA_2_ will enhance the virulence of the entomopathogens as biological control agents, as demonstrated in lepidopteran (24), coleopteran (25), and dipteran (26) pests. In this regard, PLA_2_ would be reframed as a molecular target for development of novel insecticides to synergize the microbial insecticides in *A. sapporensis* (27). Here, we report on identification of four previously unknown secretory PLA_2_s of *A. sapporensis* and their physiological functions in mediating cellular immune responses.

## Materials and methods

### Insect rearing

A field population of *A. sapporensis*, used in all experiments reported in this paper, was collected from a Welsh onion (*Allium fistulasum* L.) field (Suanbo, Korea) and reared under laboratory conditions at 27 ± 2°C, photoperiod 16:8 h (L:D) with 65 ± 5% relative humidity. Adults were kept in an acrylic cage and fed *ad libitum* with 10% sucrose solution for their diet. Fresh onion leaves were placed in the cage for egg laying and replaced daily. The egg-laid leaves were placed in a breeding dish (90 mm diameter and 15 mm height) for larval hatching. Larvae underwent five instars (L1–L5) over 16 ± 2 days in the laboratory conditions. Resulting pupae were kept at 85% relative humidity for emergence.

### Chemicals

Arachidonic acid (AA: 5,8,11,14-eicosatetraenoic acid), bromoenol lactone (BEL), and methyl arachidonyl fluorophosphate (MAFP) were purchased from Sigma-Aldrich Korea (Seoul, Korea) and dissolved in dimethyl sulfoxide (DMSO). Bromophenacyl bromide (BPB) was purchased from Tokyo Chemical (Tokyo, Japan). sPLA_2_ enzyme assay kits were purchased from Cayman Chemical (Ann Arbor, MI, USA). For RNA preparation, 1 ml of diethyl pyrocarbonate (DEPC) was combined with 1 L of deionized distilled water to prepare DEPC water and incubated for 12 h at 37°C. The water was then autoclaved twice and held at room temperature (RTP) until use. Phosphate-buffered saline (PBS) was prepared with 100 mM phosphate with 0.7% NaCl and adjusted to pH 7.4 with 1 N NaOH.

### Bioinformatics analysis

Four PLA_2_ genes were obtained from an annotated *A. sapporensis* transcriptome (GenBank accession number of PRJNA834156). They were retrieved with accession numbers OQ625511 (*As-PLA_2_A*), OQ625512 (*As-PLA_2_B*), OQ625513 (*As-PLA_2_C*), and OQ625514 (*As-PLA_2_D*). MEGA6 (28) was used to construct phylogenetic trees using the neighbor-joining method and the Poisson correction model. Bootstrap values at each branch were calculated with 1,000 repeats. InterPro (http://www.ebi.ac.uk/interpro/) was used to predict the protein domain, and the SigmalP 5.0 server (http://services.healthtech.dtu.dk/service.SignalP-5.0) was performed to detect the N-terminal signal peptide. Sequence alignment was performed using the MegAlign program (DNAStar 7.0, Lasergene, Madison, WI, USA) to get consensus residues among different PLA_2_ genes.

### RNA extraction, reverse transcriptase-polymerase chain reaction (RT-PCR), and RT-quantitative PCR (RT-qPCR)

Total RNAs were extracted from developmental stages and larval tissues indicated in Results using a Trizol reagent (Invitrogen, Carlsbad, CA, USA) according to the manufacturer’s instructions. RT-premix (Intron Biotechnology, Seoul, Korea) with the oligo-dT primer was used to synthesize complementary DNA (cDNA) from the extracted RNA (1 µg per sample) and quantified using a spectrophotometer (NanoDrop, Thermo Fisher Scientific, Wilmington, DE, USA). RT-PCR was performed using DNA Taq polymerase (GeneAll, Seoul, Korea) with an initial heat treatment at 95°C for 2 min, followed by 35 cycles of DNA denaturation at 95°C for 30 s, annealing for 1 min at varied temperatures using different gene-specific primers (**Table S1**), and chain extension at 72°C for 1 min. PCR reaction samples (25 µl) consisted of 2.5 µl of 10× Taq buffer, 2.5 µl of 10× dNTP, 1 µl of Taq DNA polymerase, each 1 µl of forward and reverse primers (10 pmol), 1 µl of template (cDNA), and 16 µl of deionized distilled water. The PCR result was validated using 1% agarose gel electrophoresis. RT-qPCR was performed using a real-time PCR system (Step One Plus Real-Time PCR System, Applied Biosystems, Singapore) in accordance with the instructions provided by (29). The reaction mixture (20 µl) included 7 µl of deionized distilled water, 10 µl of Power SYBR Green PCR Master Mix, 1 µl of cDNA template (70 ng), and each 1 µl of forward and reverse primers (**Table S1**). After activating Hot-start Taq DNA polymerase at 94°C for 5 min, the reaction was amplified with 40 cycles of denaturation at 94°C for 30 s, annealing at different temperatures (**Table S1**) for 1 min, and extension at 72°C for 30 s. A ribosomal gene, *RL32* was used to validate the cDNA integrity. Each treatment was replicated three times by preparing individual sample preparation. A comparative CT method was used to calculate the relative expression levels (30).

### Immune challenge

*Escherichia coli* Top10 was purchased from Invitrogen and grown overnight at 37°C in Luria–Bertani (LB) medium (BD, Franklin Lakes, NJ, USA). Then bacteria were heat-killed at 95°C for 10 min prior to immune challenge. For the immune challenge, *E. coli* (1.64 × 10^6^ cells/larva) was injected with a micro-capillary using a shutter CO_2_-based pico-pump injector (PV830, World Precision Instrument, Sarasota, FL, USA) under a stereomicroscope (SZX-ILLK200, Olympus, Tokyo, Japan). To determine inhibitory effects, selected chemicals or prepared dsRNA was injected along with *E. coli*. To rescue the immunosuppression, AA was coinjected with *E. coli*.

### RNA interference

A T7 promoter sequence was added to four gene-specific PLA_2_ primers at the 5’ end. Using these primers, the four As-PLA_2_ genes were amplified as just described. After amplifying all genes, the PCR products were used to prepare double-stranded RNA (dsRNA) by the Megascript RNAi Kit (Ambion, Austin, TX, USA) according to their instructions. A green fluorescence protein (*GFP*) was used as control dsRNA (dsCON). Before injection, dsRNA was combined with a transfection reagent (Metafectene Pro, Biontex, Planegg, Germany) in a 1:1 ratio. After that, 1 µg of dsRNA was injected into L5 larva as described above. RNAi efficiency was determined by RT-qPCR at time points indicated in Results. Each treatment was replicated three times with independent RNA preparations.

### Western blotting

L5 larvae were immune challenged as described. Sterilized PBS was injected as a control treatment, represented as a naïve treatment. After removing the intestine, the remaining whole body was collected from naïve or challenged insects at time points indicated in Results into 1X PBS containing a protease inhibitor cocktail (Sigma-Aldrich Korea) and phenylmethylsulfonyl fluoride (Thermo Fisher Scientific Korea, Seoul, Korea). The samples were crushed completely and centrifuged at 500 × *g* for 5 min at 4°C. After centrifugation, the supernatants were transferred to new tubes. The collected supernatants were mixed with 4× denatured sample buffer [300 mM Tris-HCL, 600 mM dithiothreitol, 12% sodium dodecyl sulfate (SDS), 0.6% bromophenol blue, and 60% glycerol]. After a 5 min heat treatment at 95°C, the extracted proteins were separated using 10% SDS-PAGE at a constant 100 V. The nitrocellulose membrane (BioRad, Hercules, CA, USA) was used to transfer the separated proteins from the gel with a transfer buffer (25 mM Tris, 190 mM glycine, 20% methanol, pH 8.5) at 100 V for 50 min at 4°C. After the membrane was washed with PBS, the background in the membrane was blocked with 5% skim milk in PBS for 1 h at RTP. After discarding the blocking solution, the membrane was again incubated with a primary antibody (diluted by 1:5,000 with 5% skim milk) raised against sPLA_2_ of *S. exigua* (Se-sPLA_2_) (31) for 3 h at RTP. After washing three times with PBS, the membrane was incubated with anti-rabbit Immunoglobulin G (IgG)–alkaline phosphatase secondary antibody (Sigma-Aldrich Korea) (dilution by 1:30,000 with 5% skim milk) for 1 h at RTP. The membrane was washed three times with PBS. Finally, nitrocellulose membrane blots were incubated for approximately 2 min with nitro blue tetrazolium/5-bromo-4-chloro-3’-indolyphosphate *p*-toluidine salt as a substrate to reveal alkaline phosphatase activity.

### Phospholipase A_2_ enzyme assay

A commercial PLA_2_ kit (Cayman Chemical, Ann Arbor, MI, USA) was used to determine the enzyme activities as detailed by Vatanparast and Kim (32). The samples were collected as just mentioned. Reaction mixtures (225 µl) were composed of 10 µl of each sample, 10 µl of Ellman’s reagent, 5 µl of assay buffer, and 200 µl of substrate (sPLA_2_). The same volume of reaction mixture consisting of 10 µl 5,5’-dithiobis 2-nitrobenzoic (DTNB), 15 µl assay buffer, and 200 µl substrate was used as a negative control. The amount of enzyme activity was measured using a spectrofluorometer (VICTOR multilabel plate reader, PerkinElmer, Waltham, MA, USA). Changes in absorbance at 405 nm of the reaction product were measured and plotted to obtain the slope of a linear portion of the curve. Enzyme activity was calculated with an extinction coefficient of DTNB (14,150 M^-1^cm^-1^). Specific enzyme activity was calculated by normalizing the spectrophotometer readings to the total amount of the protein in the samples (33). Each treatment was replicated thrice with individual sample preparations.

### Hemocyte-spreading behavior assay

L5 larvae were used for assessing the hemocyte-spreading assay. The larvae were immune-challenged as just described. To inhibit the immune response, BPB (1 µg per larva) was coinjected. At 2 and 8 h post-infection (pi), 10 µl of hemolymph from each of 15 insects was collected on a glass slide, directly fixed with 4% paraformaldehyde dissolved in PBS and incubated for 10 min at RTP under darkness. After washing three times with 1X PBS, cells were permeabilized with 0.2% Triton-X in PBS for 2 min. Again, the cells were rinsed three times with PBS and incubated with 5% skim milk for 10 min at RTP. After washing with PBS, hemocytes were incubated with 1.2% fluorescein isothiocyanate (FITC)–tagged phalloidin for 1 h at RTP. After washing three times, the cells were incubated with 4´,6-diamidino-2-phenylindole (DAPI, 1 mg/ml). After 10 min incubation, cells were washed three times with PBS and observed under a fluorescence microscope (DM2500, Leica, Wetzlar, Germany) at ×200 magnification. Each treatment was replicated three times.

### Nodulation assay

Overnight-cultured *E. coli* was used in the nodulation formation assay. Before injection, the live bacteria were killed by incubating at 95°C for 5 min and the bacterial cell number was counted using a hemocytometer (Neubauer improved bright line, Superior Marienfeld, Lauda-Konigshofen, Germany) under a phase contrast microscope (BX41, Olympus, Tokyo, Japan). L5 larvae were injected with 1 µl of *E. coli* (1.64 × 10^6^ cells/larva) along with 1 µg of BPB or dsRNA using micro-capillaries. For the control treatment, the larvae were injected with *E. coli* without adding any inhibitors or with dsCON. Then, the injected larvae were reared under laboratory conditions for 8 h. Finally, the nodules were counted by dissecting the larvae under a stereomicroscope (Stemi SV 11, Zeiss, Jena, Germany) at ×50 magnification.

### Data analysis

All experiments in this study were performed with three replicates, and a Sigma plot was used to plot the results by mean and standard deviation. Means and variances of treatments were compared by a least squared difference (LSD) test of one-way analysis of variance (ANOVA) using PROC GLM of the SAS (34) program and discriminated at Type I error = 0.05.

## Results

### Four phospholipase A_2_s predicted from *A. sapporensis* transcriptome

**Figure 1:**
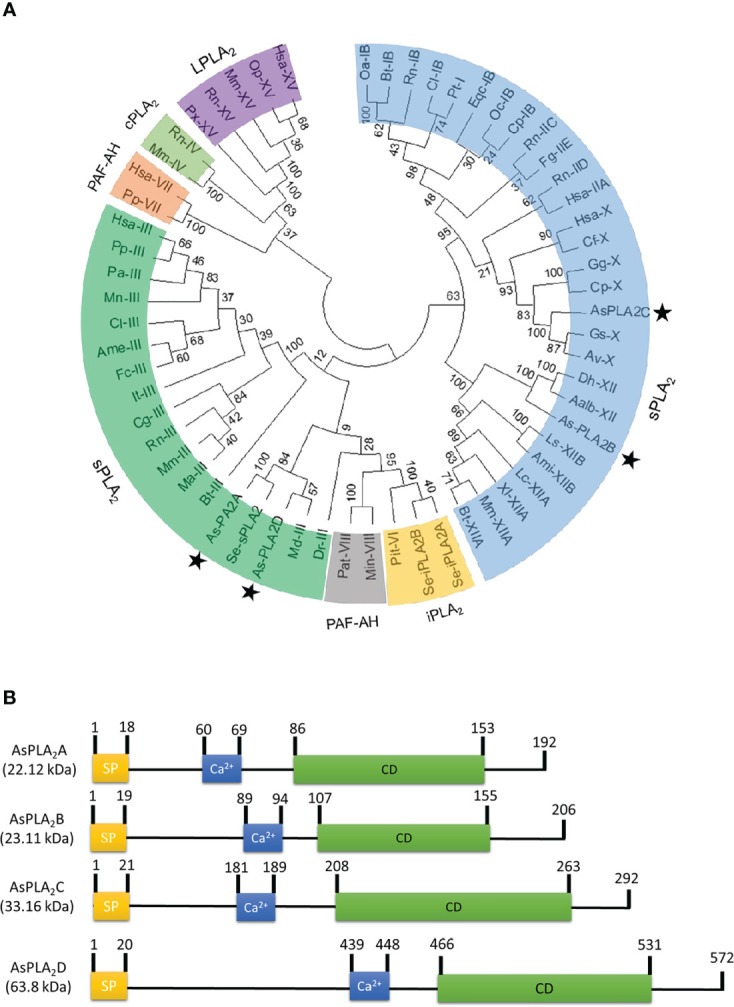
Identification of four phospholipase A_2_ (PLA_2_) genes (asterisks) from a transcriptome of *A. sapporensis*. **(A)** Phylogenetic tree of different PLA_2_s including four As-PLA_2_s using the MEGA6 program. Bootstrap values were obtained with 1,000 repetitions to support branching and clustering. Different types of PLA_2_s include secretory phospholipase A_2_ (sPLA_2_), calcium-independent phospholipase A_2_ (iPLA_2_), cytosolic phospholipase A_2_ (cPLA_2_), lysosomal phospholipase A_2_ (LPLA_2_), and platelet-activating factor acetyl hydrolase (PAF-AH). **(B)** Comparison of functional domains of four *A. sapporensis* sPLA_2_ (*As-sPLA_2_*) genes. ‘SP’, ‘Ca^2+^’, and ‘CD’ stand for signal peptide, calcium-binding site, and catalytic domain. InterPro (http://www.ebi.ac.uk/interpro/), Expasy (www.expasy.com) and SigmalP 5.0 server (http://services.healthtech.dtu.dk/service.SignalP-5.0) were used to predict domain and signal peptides. Molecular weights of the four As-PLA_2_s were predicted using the EditSeq program of DNAStar 7.0. The numbers above domains indicate the locations of amino acids.

Transcriptome analysis annotated four PLA_2_ genes: *As-PLA_2_A*, *As-PLA_2_B*, *As-PLA_2_C*, and *As-PLA_2_D* (**Figure 1**). A phylogenetic analysis with 11 known PLA_2_s groups (**Figure 1A**) included four PLA_2_ types: sPLA_2_, iPLA_2_, cPLA_2_, and PAF-AH. In this classification, the four *A. sapporensis* PLA_2_s were clustered with other sPLA_2_s. Specifically, *As-PLA_2_A* and *As-PLA_2_D* were assigned into Group III, *As-PLA_2_B* into Group XII and *As-PLA_2_C* into Group X. These four PLA_2_s were then compared in their functional domains using a proteomics bioinformatics tool (www.expasy.com). Each PLA_2_ has a separate signal peptide representing secretory proteins (**Figure 1B**). They also share calcium binding sites with a conserved glycine residue (**Figure S1**) indicating their dependence on Ca^2+^. In the catalytic domains, a histidine-aspartic acid (‘HD’, **Figure S1**) dyad occurred in all four PLA_2_s. Finally, more than 12 cysteine residues were encoded in the four PLA_2_s.

### Expression profile of four phospholipase A_2_s

**Figure 2:**
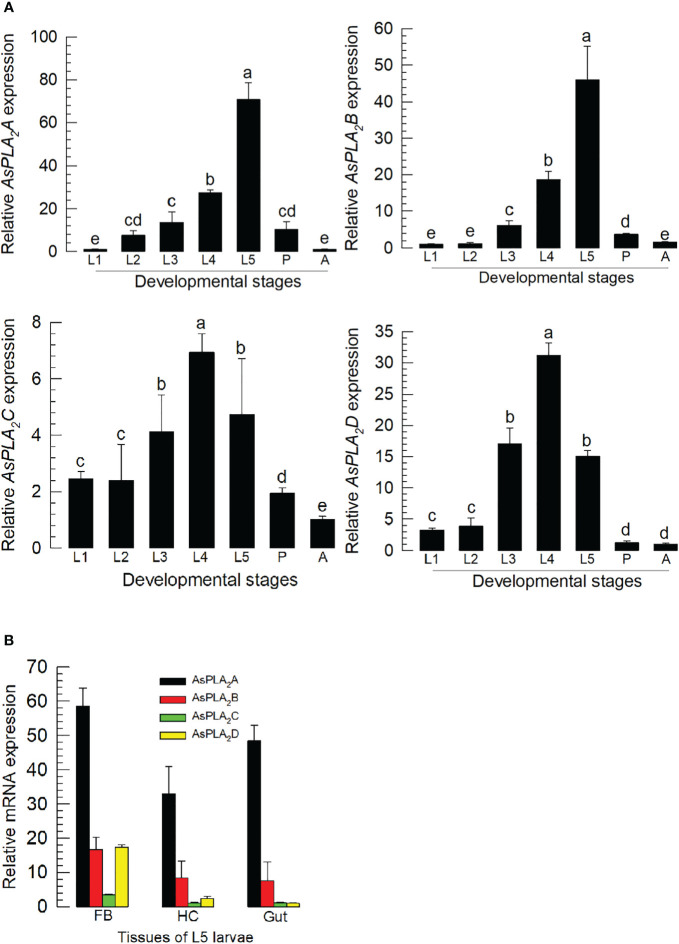
Expression profile of PLA_2s_ in *A. sapporensis*. **(A)** Expression pattern of *As-PLA_2_A*, *As-PLA_2_B*, *As-PLA_2_C*, and *As-PLA_2_D* in whole body samples of different developmental stages: first to fifth instar larva (‘L1–L5’), pupa (‘P’), and adult (‘A’). Different letters above standard deviation bars indicate significant differences among means at Type I error = 0.05 [least squared difference (LSD) test]. **(B)** PLA_2_ expression analysis in different tissues of L5 larvae including the fat body (‘FB’), hemocyte (‘HC’), and gut. A ribosomal gene, *RL32*, was used as a reference gene. Each treatment was replicated three times with independent sample preparations.

All four PLA_2_s were expressed through development from larva to adult stages (**Figure 2A**). Their expression levels increased with larval development and showed the highest expressions at the final larval instar for *As-PLA_2_A* and *As-PLA_2_B* and at the penultimate instar for *As-PLA_2_C* and *As-PLA_2_D*. In the larval stage, the four PLA_2_s were expressed in all the tested tissues (hemocyte, fat body, and gut) with high expression at the fat body (**Figure 2B**). Among the four PLA_2_s, *As-PLA_2_A* was highly expressed in all tissues.

### Phospholipase A_2_ enzyme activity

**Figure 3:**
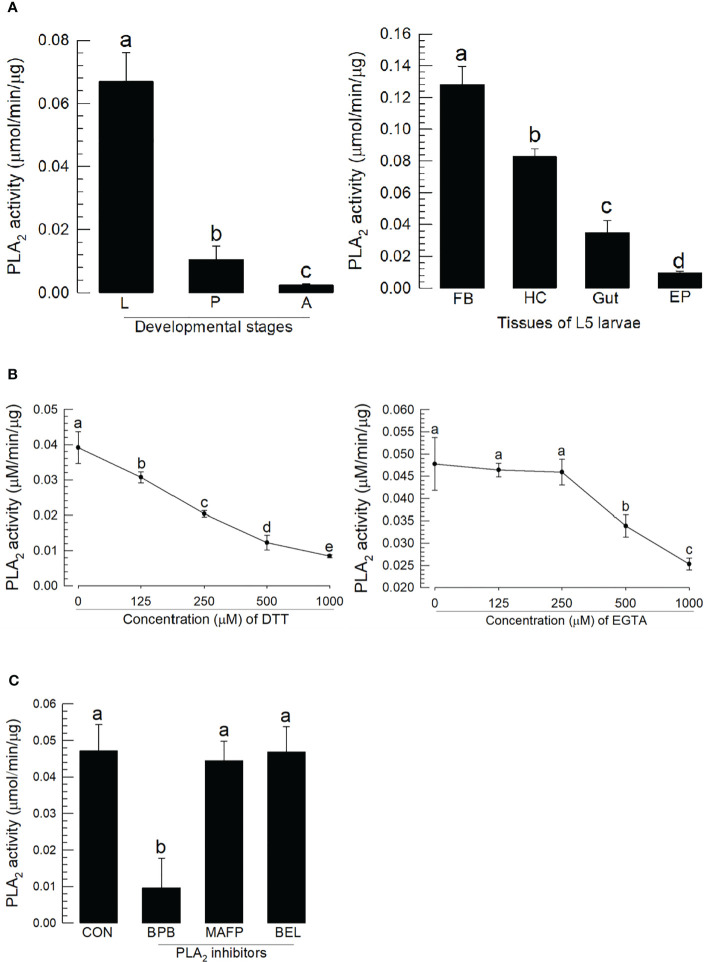
Characterization of *A. sapporensis* PLA_2_ enzyme activity. **(A)** Differential specific enzyme activities in developmental stages and larval tissues: larva (‘L’), pupa (‘P’), adult (‘A’), fat body (‘FB’), hemocyte (‘HC’), gut, and epidermis (‘EP’). **(B)** Inhibitory effects of dithiothreitol (DTT) and ethylene glycol-bis(β-aminoethyl ether)-N,N,N′,N′-tetraacetic acid (EGTA) on the PLA_2_ enzyme activity. The enzyme was extracted from the whole body of L5 larvae after removing the gut. **(C)** Influence of different PLA_2_ inhibitors [bromophenacyl bromide (BPB), bromoenol lactone (BEL), and methyl arachidonyl fluorophosphate] on the PLA_2_ enzyme activity. Different letters above standard deviation bars indicate significant differences among means at Type I error = 0.05 (LSD test).

PLA_2_ activity was monitored across developmental stages (**Figure 3A**). As expected, larval stage showed higher specific enzyme activity compared to pupal and adult stages. The larval fat body also expressed the highest enzyme activity compared to hemocytes, gut, and epidermis.

We monitored the disulfide linkage and its influence on the catalytic activity, by running enzyme assays in the presence of dithiothreitol, which inhibited the enzyme activity in a dose-dependent manner (**Figure 3B**). We monitored Ca^2+^ dependence, by running assays in the presence of ethylene glycol-bis(β-aminoethyl ether)-N,N,N′,N′-tetraacetic acid (EGTA), which led to a dose-dependent inhibition of the activity. Three inhibitors specific to different types of PLA_2_s were applied to the reaction mixture (**Figure 3C**). BPB, a sPLA_2_-specific inhibitor, inhibited the PLA_2_ activity as expected; however, reactions in the presence of BEL (a iPLA_2_-specific inhibitor) or MAFP (a cPLA_2_-specific inhibitor) did not influence enzyme activity.

### Induction of phospholipase A_2_s expression and enzyme activity in response to immune challenge

**Figure 4:**
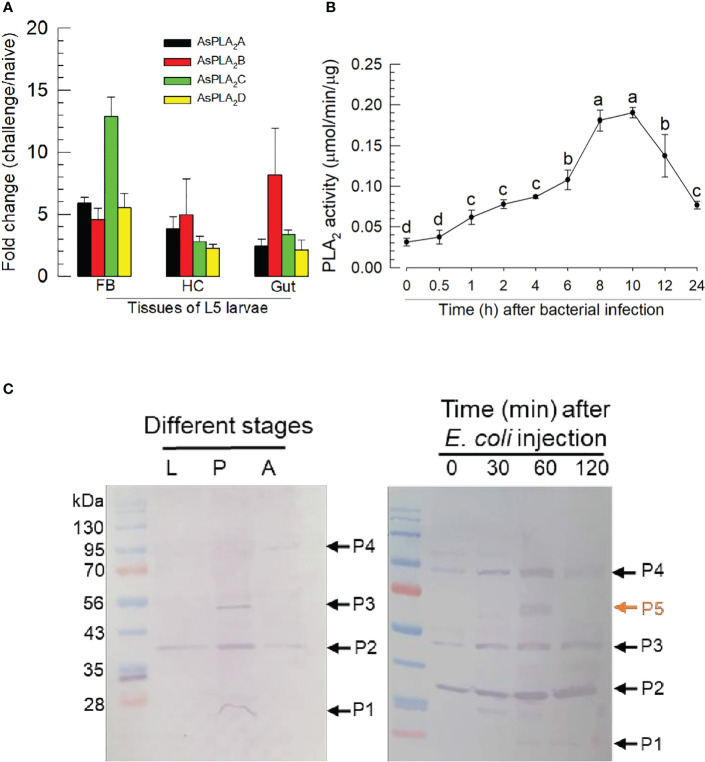
Inducible PLA_2_ enzyme activity in *A. sapporensis* larvae after immune challenge. **(A)** Inducible expressions of four *As-PLA_2_* genes after the immune challenge in different larval tissues: fat body (‘FB’), hemocyte (‘HC’), and gut. **(B)** Induced PLA_2_ activities after the immune challenge. Enzyme was extracted from the whole body of L5 larvae after removing the gut. **(C)** Western analysis of As-PLA_2_s in naïve and challenged larvae. Three developmental stages (left panel) used naïve insects. After the immune challenge (right panel), the gut-removed whole body to avoid any other PLA_2_s in the gut lumen was used for the immunoblotting analysis with a polyclonal antibody raised against *S. exigua* sPLA_2_. Here, ‘0 min’ sample represents just before the immune challenge. Each lane contained 50 μg proteins. ‘P1–P5’ indicate five protein bands detected by the Western blot analysis. Each qPCR or enzyme assay was replicated three times with independent sample preparations. Different letters above standard deviation bars indicate significant differences among means at Type I error = 0.05 (LSD test).

Challenge by injecting a non-pathogenic bacterium, *E. coli*, led to more than twofold increases in expression of the PLA_2_ genes in fat body, hemocyte, and gut (**Figure 4A**). The gene encoding *As-PLA_2_C* was the most highly induced in fat body by almost 13-folds while other PLA_2_s showed approximately 5-fold induction. This induction of the gene expressions was further supported by the increase of the enzyme activity, in which there was an approximate sixfold increase in PLA_2_ activity over the following 8~10 h (**Figure 4B**). As early as 1 h postinjection (pi), PLA_2_ activity was highly induced and kept the increase mode up to 8 h pi. The induced enzyme activity was decreased after 10 h pi. To visualize the PLA_2_s of *A. sapporensis*, a Western blot analysis was conducted using a polyclonal antibody raised against Se-sPLA_2_ of another lepidopteran. *S. exigua* (**Figure 4C**). *As-PLA_2_A* and *As-PLA_2_D* share sequence homologies (38.4%–53.6%) with that of Se-sPLA_2_, while *As-PLA_2_B* and *As-PLA_2_C* exhibit relatively low homologies (9.8%–11.0%) (**Figure S2**). The Western analysis showed four bands at approximately 25 kDa (‘P1’), 38 kDa (‘P2’), 55 kDa (‘P3’), and 90 kDa (‘P4’). After the immune challenge, an additional band (‘P5’) were detected at approximately 65 kDa.

### Cellular immune responses influenced by phospholipase A_2_

**Figure 5:**
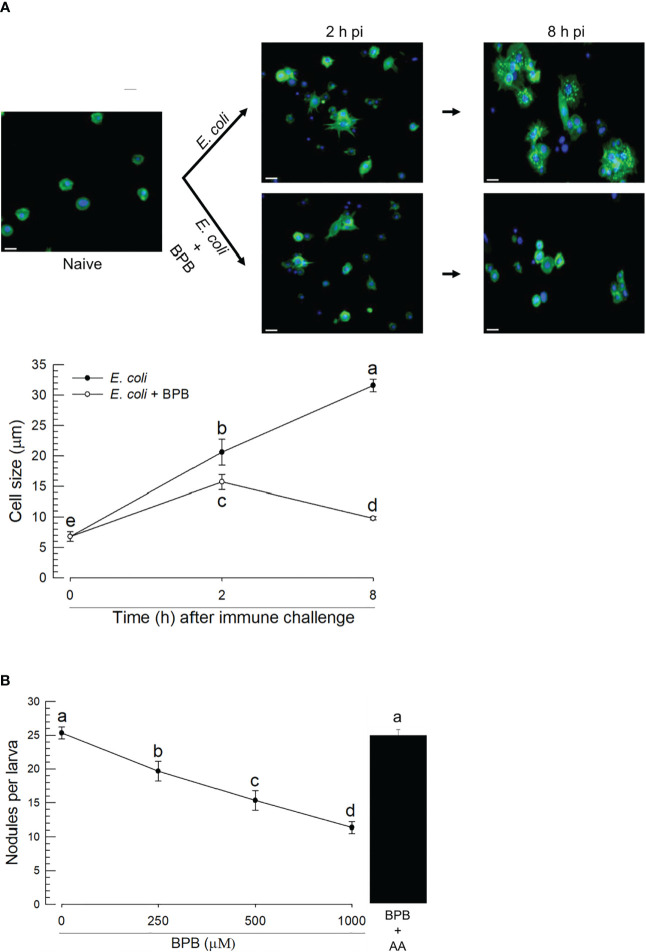
Functional association of PLA_2_ activity with cellular immune responses in *A. sapporensis*. To inhibit PLA_2_ activity, BPB (1 μg per larva) was injected to the hemocoel of L5 larvae. **(A)** Influence of BPB on hemocyte-spreading behavior at 2 and 8 h after the immune challenge. The white scale bars represent 10 μm. Hemocyte cell size represents the longest cell diameter on the image. Each measurement used randomly chosen 30 hemocytes and was replicated three times with independently prepared hemocyte samples. **(B)** Inhibitory effect of BPB on nodule formation after the immune challenge. After 8 h pi with immune challenge along with BPB, the larvae were dissected to evaluate the number of nodules. Only *E. coli* was injected for a control treatment. AA (arachidonic acid, 1 μg per larva) was injected along with BPB. Different letters above the error show significant differences among means at Type I error = 0.05 (LSD test).

The upregulations of PLA_2_ gene expression and enzyme activity upon the bacterial infection, suggesting its physiological role in immune responses. Hemocytes were spread after the immune challenge as early as 2 h pi and kept the spread behavior 8 h pi (**Figure 5A**). In contrast, BPB injection along with the immune challenge suppressed the hemocyte behavior. As this hemocyte behavior is required for cellular immune responses, we assessed nodule formation of the hemocytes against the immune challenge. In control larvae, approximately 25 nodules were formed after the bacterial injection at 8 h pi (**Figure 5B**). However, the BPB addition significantly prevented this nodule formation in a dose-dependent manner. Interestingly, the addition of arachidonic acid (a catalytic product of PLA_2_) significantly rescued the inhibitory activity of BPB.

### Individual RNA interference treatments of four phospholipase A_2_s suppress the cellular immune response

**Figure 6:**
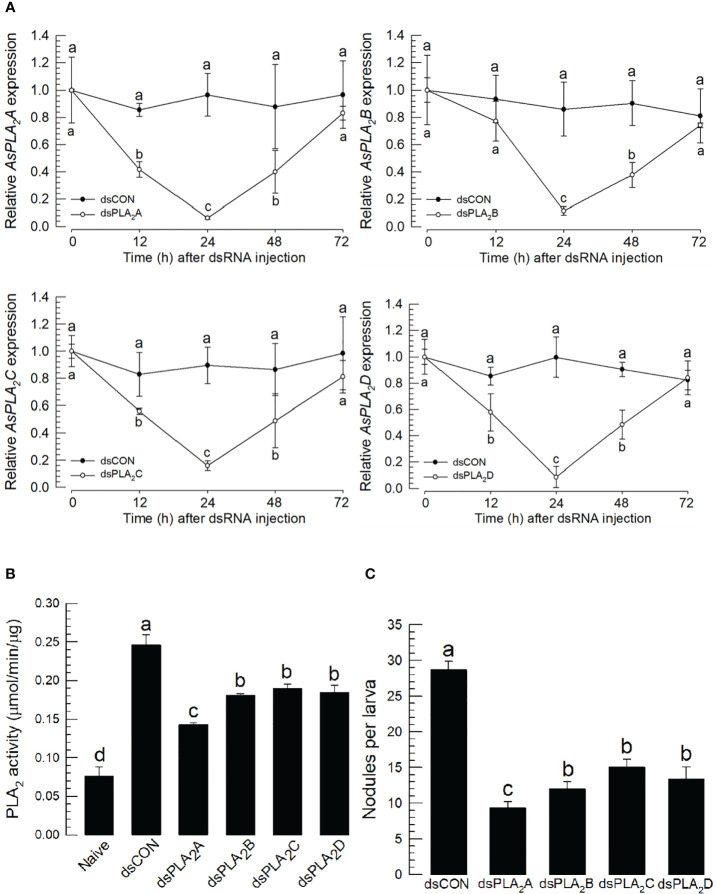
Functional assay of each *As-sPLA_2_* in mediating cellular immune response in *A. sapporensis*. Individual RNA interference (RNAi) treatments were applied by injecting 1 μg of dsRNA (‘dsPLA_2_A–dsPLA_2_D’) to L5 larva. A green fluorescence protein was used as a control dsRNA treatment (‘dsCON’). **(A)** Reduction of specific *As-sPLA_2_* genes by individual RNAi treatments. **(B)** Suppression of PLA_2_ activity induced by the immune challenge by the individual RNAi treatments. **(B)** Influence of the individual RNAi treatments on nodule formation. Each treatment was replicated thrice with independent sample preparations. Different letters above the standard deviation bars show significant differences among means at Type I error = 0.05 (LSD test).

To determine which sPLA_2_(s) of *A. sapporensis* might be associated with the cellular immune responses, individual RNAi treatments were applied to each of four *As-sPLA_2_* genes (**Figure 6**). Injection of dsRNAs specific to each of *As-PLA_2_* genes significantly suppressed their target genes for at least 48 h pi (**Figure 6A**). Under these individual RNAi conditions, PLA_2_ enzyme activities were monitored after immune challenge (**Figure 6B**). As expected, the immune challenge significantly induced the enzyme activity. However, all the four individual RNAi treatments significantly suppressed the induction of the enzyme activity after the immune challenge. The four individual RNAi treatments then inhibited the nodule formation after the immune challenge (**Figure 6C**).

## Discussion

Here, we report identification of four newly discovered genes encoding PLA_2_s in *A. sapporensis* and lay out an argument that these genes can be developed into targets that will effectively cripple insect immune reactions to infections. First, based on a field study conducted in Turkish fields surrounding Kahramanmaraş, Tunaz and Stanley (35) reported that 98% of all collected insect specimens either were or had been infected by naturally occurring microbes, indicated by the presence of melanized nodules in their hemocoels. These data document the need and deployment of effective innate immunity in insect biology. Second, phylogenic and domain analyses of the genes encoding PLA_2_s document identification of the genes. Third, qPCR analysis shows the genes are most highly expressed in larvae and, within larvae, the genes are expressed in the fat body, hemocytes, and alimentary canal. Fourth, our data show that the enzymes encoded by the four genes are sensitive to the expected inhibitory conditions for PLA_2_s. Fifth, bacterial infection led to increased expressions of genes encoding the four PLA_2_s and in PLA_2_ enzyme activity. Sixth, treating larvae with inhibitors of PLA_2_ activity led to reductions in two specific immune actions: hemocyte spreading and nodulation. While these points are not a comprehensive summary, they amount to a strong argument that PLA_2_s play crucial roles in mediating immune responses in *A. sapporensis* and possibly other pest insect species

The PLA_2_ activity reported here can be inhibited by a sPLA_2_-specific inhibitor, but not by iPLA_2_- or cPLA_2_-inhibitors, indicating that is it a sPLA_2_. All four PLA_2_s are secretory enzymes assigned to three sPLA_2_ groups (III, X, and XII). PLA_2_s are classified into 16 groups based on amino acid sequences; they include sPLA_2_, iPLA_2_, and cPLA_2_ (7). In this classification, Group III sPLA_2_s consists of PLA_2_s derived from insects and divided into two subgroups (venomous and non-venomous) (36). The lepidopteran, *S. exigua*, expresses a single sPLA_2,_ *Se-sPLA_2_*, classified into Group III. This enzyme mediates immune responses (31). *As-PLA_2_A* and *As-PLA_2_D* are closely clustered with *Se-sPLA_2_* in our phylogenetic analysis, from which we suggest that they act in mediating immune responses. *As-PLA_2_B* is assigned into Group XII. Another Group XII PLA_2_ identified from a hemipteran *Rhodnius prolixus* produces eicosanoids via releasing PUFAs from cellular PLs (37). In a mosquito, *Aedes albopictus*, four PLA_2_s including Group XII are identified and speculated to be associated with biosynthesis of PGE_2_ for oogenesis (38). PGE_2_ also acts in mediating insect immune responses (15). This also suggested that *As-PLA_2_B* acts in mediating immune responses. We assigned *As-PLA_2_C* into Group X in our phylogenetic analysis, the first insect PLA_2_s classified into this group.

The expression profiles of these *As-sPLA_2_* genes indicates changing PLA_2_ enzyme activities during immature development. Gene expressions increased during larval development, with highest expression and enzyme activity during late larval instars. Insect sPLA_2_ also act in lipid digestion by producing lysophospholipids, which are presumed to act like mammalian bile because insects do not produce bile (39). The increase of the PLA_2_ activity reported here may be consistent with increased feeding activity during larval development. The correlation between gene expression and the enzyme activity was also recorded after the immune challenge. Expression of the As-sPLA_2_ genes was highly induced after immune challenge, which may have led to significant increases in PLA_2_ enzyme activities. Based on the high sequence similarities between As-sPLA_2_s and Se-sPLA_2_, we performed a western analysis to assess the change in PLA_2_ protein. Naïve larvae showed four bands (‘P1–P4’), in which the P1 protein band matched the predicted molecular weights of *As-PLA_2_A* or *As-PLA_2_B* while P2 appeared to match with the size of *As-PLA_2_C*. However, protein bands at 55 kDa (‘P3’) and 90 kDa (‘P4’) were not explained by current four PLA_2_s. In contrast, the protein samples extracted from immune-challenged larvae had an additional band (‘P5’) that matched the predicted molecular weight of *As-PLA_2_D*. Ongoing research will clarify these points.

PLA_2_ activity mediates *A. sapporensis* cellular immune responses. We recorded hemocyte spreading, a visible immune reaction to infections that was inhibited in larvae treated with an inhibitor of PLA_2_ activity. Hemocyte spreading is a phase of cellular immune actions including phagocytosis, nodulation, and encapsulation (40). The spreading involves cytoskeleton rearrangement and local volume change, which are mediated by eicosanoids (41). Influencing cytoskeletons may be a common PG function in animal cells. Green et al. (42) reported earlier that cytoskeleton components are also specific targets of PG signaling in *Drosophila* egg development. As already mentioned, PLA_2_ is the first step in eicosanoid biosynthesis and inhibiting this enzyme likely inhibits such subcellular actions generally.

Individual RNAi treatments suppressed their target mRNA levels for at least 48 h pi. Our immune assays were performed at 24 h pi, when the RNAi effects were maximal for all four PLA_2_s. The RNAi efficacies are supported by the decreases in PLA_2_ activities. Under these RNAi conditions, the cellular immune response, nodulation, was suppressed following the four RNAi treatments. Our interpretation is that each of the four PLA_2_s is associated with cellular immune responses. We suggest that each of these four enzymes is required for cellular immune responses because RNAi treatment against one of the four PLA_2_s significantly suppressed nodulation. Multiple PLA_2_s forms are expressed in specific mammalian tissues. Rat brain expresses six PLA_2_s including four sPLA_2_s, an iPLA_2_, and cPLA_2_ in different regions (43), which may be required for regulating synaptic plasticity. Here, we speculate that the four *A. sapporensis* PLA_2_s may be expressed in different hemocyte subtypes of hemocytes and they may act in hemocyte-specific immune responses. For example, two cell types, granulocytes and plasmatocytes, form multicell layers around pathogens and a third type, oenocytoids, releases prophenoloxidase during nodule formation (40, 44).

## Data availability statement

The datasets presented in this study can be found in online repositories. The names of the repository/repositories and accession number(s) can be found in the article/**Supplementary Material**.

## Author contributions

MH: methodology, resources, software, formal analysis, investigation, writing—original draft, and visualization. JH: methodology, resources, software, formal analysis, and investigation. YK: conceptualization, methodology, project administration, writing—original draft, writing—review and editing, and funding acquisition. All authors contributed to the article and approved the submitted version.
