# Supplementary material for: Identification of four secretory phospholipase A2s in a lepidopteran insect, Acrolepiopsis sapporensis, and their functional association with cellular immune responses
Source: Front Endocrinol (Lausanne). 2023 Jun 23;14:1190834. doi: 10.3389/fendo.2023.1190834 (PMC10328117; doi:10.3389/fendo.2023.1190834)
Supplement: Supplementary file 1 [file DataSheet_1.docx]

**Supplementary Information**

**Table S1.** Primers used in this study

**Fig. S1.** Alignment of the specific region of the PLA_2_s sequences. (A) Alignment of the calcium-binding site of PLA_2_A, PLA_2_B, PLA_2_C, and PLA_2_D. Two asterisks represent the glycine residues. (B) Alignment of the catalytic site (CD) specific region. The conserved active site (His-Asp dyad) was indicated by two arrows.

**Fig. S2.** Comparison of four PLA_2_s (PLA_2_A, PLA_2_B, PLA_2_C, and PLA_2_D) sequences with *S*. *exigua* PLA_2_ sequence. The table shows the percent of identity and divergence respectively. Sequence alignment was performed by the Clustal W program of MegAlign (DNASTAR, Version 7.0).

**Table S1.** Primers used in this study

| Genes | Orientation | Sequence (5ʹ - 3ʹ) | Uses | Annealing temperature (ºC) |
| --- | --- | --- | --- | --- |
| AsPLA_2_A | Forward | ACAACGATATCGGCTCGTCC | RT-PCR  RT-qPCR | 53.0 |
|  | Reverse | AGCCGGTGATAGGGAAGTCT |  |  |
| T7+ AsPLA_2_A | Forward | TAATACGACTCACTATAGGGAGA ACAACGATATCGGCTCGTCC | RNAi | 57.0 |
|  | Reverse | TAATACGACTCACTATAGGGAGA AGCCGGTGATAGGGAAGTCT |  |  |
| AsPLA_2_B | Forward | GAAGGTTCGAAACCTGTCCG | RT-qPCR | 52.0 |
|  | Reverse | TATTTCCTTGCTGGTGGGCAA |  |  |
| T7+ AsPLA_2_B | Forward | TAATACGACTCACTATAGGGAGA  GAAGGTTCGAAACCTGTCCG | RNAi | 53.5 |
|  | Reverse | TAATACGACTCACTATAGGGAGA  TATTTCCTTGCTGGTGGGCAA |  |  |
| AsPLA_2_C | Forward | TAAGGGCTACGGGTGCTACT | RT-PCR | 52.0 |
|  | Reverse | TCCCCTCGATAGCACTTCCA |  |  |
| T7+ AsPLA_2_C | Forward | TAATACGACTCACTATAGGGAGA  TAAGGGCTACGGGTGCTACT | RNAi | 54.0 |
|  | Reverse | TAATACGACTCACTATAGGGAGA  TCCCCTCGATAGCACTTCCA |  |  |
| AsPLA_2_D | Forward  Reverse | CACGACGGGAGAACCACATT ACAACTCTTTCGGCCACACT | RT-qPCR | 55.0 |
| T7+ AsPLA_2_D | Forward    Reverse | TAATACGACTCACTATAGGGAGA  CACGACGGGAGAACCACATT TAATACGACTCACTATAGGGAGA  ACAACTCTTTCGGCCACACT | RNAi | 55.0 |
| A ribosomal protein L32 | Forward | ATGCCCAACATTGGTTACGG | RT-PCR  RT-qPCR | 52.0 |
|  | Reverse | TTCGTTCTCCTGGCTGCGGA |  |  |

**(A)**

**
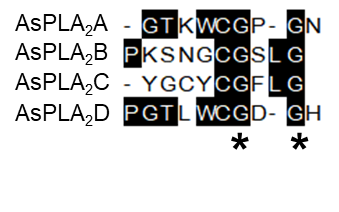
**

**(B)**

**
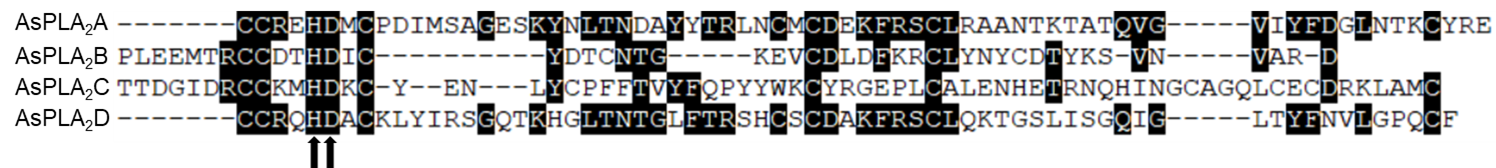
**

**Fig. S1**

**
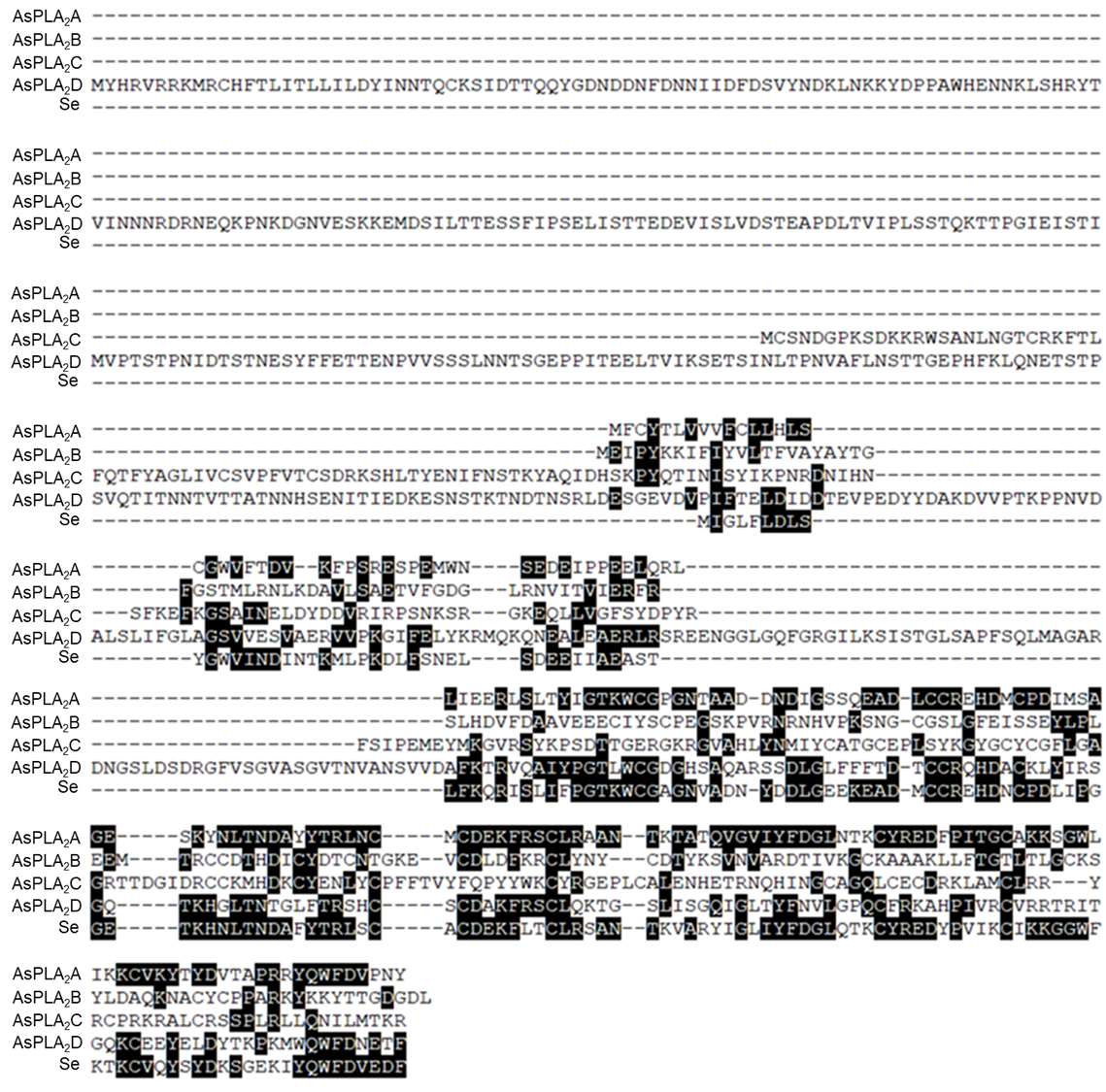
**

**
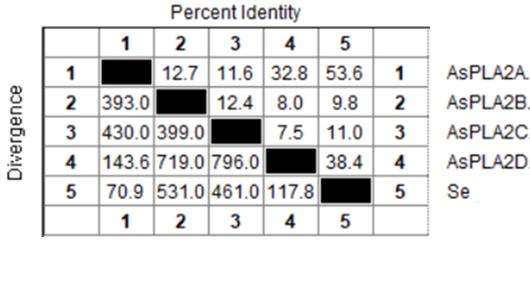
**

**Fig. S2**
